# Beyond Joints: Neuropsychiatric Benefits of TNF-α and IL-6 Inhibitors in Rheumatoid Arthritis—Narrative Review

**DOI:** 10.3390/ijms26178361

**Published:** 2025-08-28

**Authors:** Hanna Siuchnińska, Alina Minarowska, Eliza Wasilewska

**Affiliations:** 1 Occupational Therapy Center in Czersk ul Pomorska 10, 89-650 Czersk, Poland; 2 Department of Pulmonology, School of Public Health, Collegium Medicum, University of Warmia and Mazury in Olsztyn, Jagiellońska 78, 10-357 Olsztyn, Poland; 3 Department of Pediatrics, Gastroenterology, Hepatology, Nutrition, Allergology and Pulmonology, Outpatient Cystic Fibrosis Clinic, Children’s University Hospital in Bialystok, 15-274 Bialystok, Poland; 4 Department of Allergology, Medical University of Gdańsk, Smoluchowskiego 17, 80-211 Gdańsk, Poland

**Keywords:** rheumatoid arthritis, TNF-α inhibitors, IL-6 inhibitors, depression, psychiatric symptoms, neuropsychiatric symptoms, biologics, quality of life, neuroinflammation, neuroimmunology, neuroimmune modulation, blood–brain barrier, biological therapies

## Abstract

Rheumatoid arthritis (RA) is a systemic autoimmune disease that, beyond joint destruction, contributes to neuropsychiatric symptoms such as depression, anxiety, and cognitive impairment. These symptoms are often underrecognized despite their major impact on quality of life. Accumulating evidence suggests that pro-inflammatory cytokines, particularly tumor necrosis factor alpha (TNF-α) and interleukin-6 (IL-6), play a key role in this neuroimmune interface. This narrative review examined 16 clinical studies evaluating the effects of biologic therapies targeting TNF-α and IL-6 on mental health outcomes in RA. The total study population comprised 9939 patients, including 2467 treated with TNF-α inhibitors and 7472 with IL-6 or IL-6 receptor inhibitors. TNF-α inhibitors were associated with improved depressive symptoms and emotional well-being. IL-6 inhibitors demonstrated similar psychiatric benefits, particularly in patients with elevated IL-6 levels. The findings highlight that biological therapies in RA may influence not only physical symptoms but also mental health, likely through modulation of neuroimmune pathways including blood–brain barrier permeability, microglial activation, and HPA axis regulation. Future research is needed to clarify these effects in populations stratified by psychiatric comorbidity and inflammatory biomarkers. Clinical implications: Incorporating psychiatric symptom screening and considering neuroinflammatory profiles may help guide the selection of biologic therapy in RA, particularly in patients with comorbid depression or fatigue.

## 1. Introduction

Rheumatoid arthritis (RA) is a chronic, systemic autoimmune disease characterized by persistent synovial inflammation, progressive joint destruction, and extra-articular manifestations. It affects approximately 0.5% to 1% of the general population worldwide, with a higher prevalence among women and individuals in middle age [1,2]. Beyond its well-established musculoskeletal symptoms, RA has a significant impact on patients’ overall health, quality of life, and psychological well-being.

In recent years, RA has been increasingly recognized not only as a joint-specific condition but as a systemic disease involving complex interactions between the immune system, neuroendocrine pathways, and the central nervous system [3,4]. Psychiatric comorbidities, especially depression and anxiety, are frequent in patients with RA. Epidemiological studies indicate that up to 40% of RA patients experience clinically significant depressive symptoms during the course of the disease [4,5]. Depression in RA is associated with worse disease outcomes, higher levels of perceived pain, increased disability, poorer treatment adherence, and even increased mortality [6,7].

The link between systemic inflammation and psychiatric symptoms (depression, fatigue, cognitive dysfunction) is increasingly supported by both experimental and clinical data. Pro-inflammatory cytokines such as TNF-α, IL-1β, IL-6, and IL-17, which play central roles in the pathogenesis of RA, are also implicated in the development of mood disorders [8,9,10]. These cytokines can cross the blood–brain barrier or signal through peripheral nerves, activating microglia and astrocytes, and disrupting neurotransmitter systems and hypothalamic–pituitary–adrenal (HPA) axis regulation [11,12]. Such interactions lead to behavioral changes often described as sickness behavior syndrome, which overlaps with the symptomatology of depression, including fatigue, anhedonia, cognitive dysfunction, and reduced motivation [13,14].

**Figure 1:**
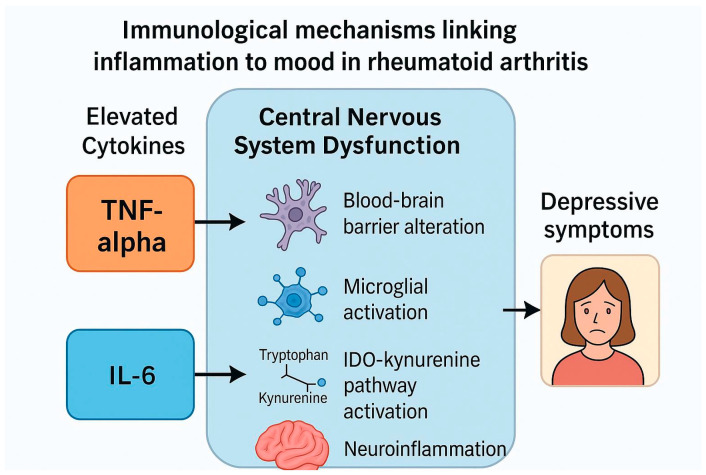
Immunological mechanism linking chronic elevated pro-inflammatory cytokines to depressive symptoms. Systemic inflammation driven by TNF-α and IL-6 contributes to neuroinflammation via blood–brain barrier disruption, microglial activation, and HPA axis dysregulation, resulting in depressive symptoms, fatigue, and cognitive dysfunction.

In the context of RA, the bi-directional relationship between chronic inflammation and mental health becomes particularly relevant. Effective immunomodulatory treatments targeting TNF-α and IL-6 not only reduce joint inflammation but may also exert beneficial effects on mood and quality of life [15,16]. These observations raise important questions about the underlying mechanisms connecting the immune system and psychiatric symptoms, and whether such effects could be harnessed in clinical practice to improve outcomes for RA patients (Figure 1).

Currently, several classes of biological therapies are available for the treatment of RA, each targeting different key components of the immune system. These include: TNF-α inhibitors (infliximab—IFX, etanercept—ETA, adalimumab—ADA, certolizumab pegol—CZP, golimumab—GLM) [2], IL-6 inhibitors (tocilizumab—TCZ, sarilumab—SAR) [17,18], B-cell depleting agents (rituximab, targeting CD20-positive B cells) [19], and T-cell co-stimulation inhibitors (abatacept, targeting CD80/CD86 via CTLA-4) [20].

Each of these drug classes acts on a distinct immunological pathway contributing to RA pathogenesis. However, among the various biological therapies approved for RA, those targeting TNF-α and IL-6 are of particular interest in the context of mental health outcomes, including neuroinflammatory and neuroendocrine alterations [21,22]. Table 1 presents the biological agents targeting TNF-α and IL-6, along with their molecular structures and immunological functions, such as cytokine neutralization or receptor blockade.

This narrative review aims to summarize current findings on the impact of TNF-α and IL-6 inhibitors on mental health outcomes, particularly depressive symptoms, in patients with RA.

## 2. Methods

The narrative review was conducted through a literature search using PubMed, Scopus, and Google Scholar, focusing on articles published between January 2000 and December 2024. Search terms included combinations of: “rheumatoid arthritis”, “biological therapy”, “TNF-α inhibitors”, “IL-6 inhibitors”, “depression”, “quality of life”, “patient-reported outcomes”, and “psychiatric symptoms”, and/or “depression” and/or “fatigue” and/or “cognitive dysfunction”.

Randomized controlled trials (RCTs), non-interventional cohort studies, post hoc analyses of clinical trials, and relevant preclinical studies were selected for inclusion. Articles were included if they reported outcomes related to mood disorders (e.g., depression, anxiety, anhedonia) or broader quality of life measures (e.g., PROMIS, SF-36, EQ-5D) in patients undergoing treatment with TNF-α or IL-6 inhibitors. Only studies published in English were considered. No systematic review methods (e.g., PRISMA guidelines) were applied, in keeping with the narrative nature of this review.

An overview of the molecular structure, pharmacological classification, and mechanisms of action of biologic agents targeting TNF-α and IL-6 used in RA is presented in Table 1. This classification includes fully human monoclonal antibodies, chimeric antibodies, humanized antibodies, receptor fusion proteins, and Fab fragments conjugated with polyethylene glycol (PEG). The table also lists commercial trade names and available biosimilars. Only biologic agents directly affecting TNF-α and IL-6 pathways are included, given their relevance to both inflammatory and neuropsychiatric outcomes discussed in this review.

## 3. Results

**Figure 2:**
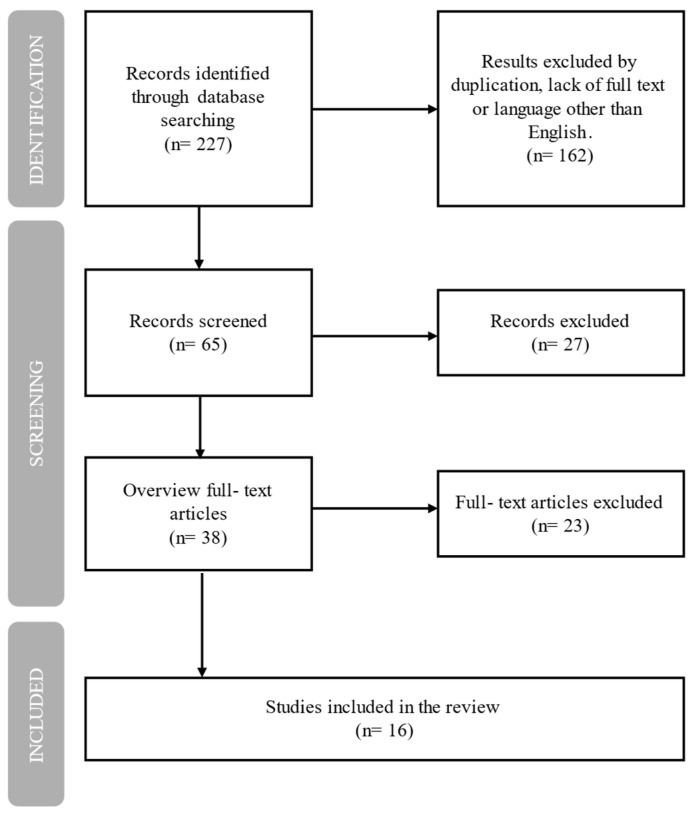
Flowchart of study selection. Diagram illustrating the process of identifying and selecting studies included in this review.

Finally, a total of 16 studies were included in the analysis. They cover both TNF-α inhibitors and IL-6/Il-6R inhibitors in relation to mental health outcomes in patients with RA (Figure 2). Across all included studies, a total of 10,499 patients were analyzed—3027 in studies evaluating TNF-α inhibitors and 7472 in studies on IL-6 or IL-6R inhibitors. Among them, approximately 6820 individuals received active treatment with biological agents: around 2211 with TNF-α inhibitors and 4609 with IL-6/IL-6R inhibitors. These patients were treated either with biological monotherapy or in combination with methotrexate (MTX), and compared to placebo, MTX alone, or standard disease-modifying anti-rheumatic drugs (DMARDs). Regardless of the combination, the presence of the biological agent was the key factor of interest in evaluating potential neuropsychiatric effects.

### 3.1. Assessment Tools Used to Evaluate Neuropsychiatric Outcomes

The included studies employed a wide range of instruments to assess neuropsychiatric symptoms and related outcomes in patients with RA (Table 2). Validated psychiatric tools such as the Hospital Anxiety and Depression Scale (HADS), Beck Depression Inventory-II (BDI-II), Hamilton Depression Rating Scale (HAM-D/HDRS), and Self-Rating Depression Scale (SDS) were applied in several trials, allowing for specific and reliable evaluation of depressive and anxiety symptoms.

In addition, some studies incorporated domains from the Patient-Reported Outcomes Measurement Information System (PROMIS), including depression, fatigue, pain interference, and physical function. PROMIS instruments provide a modern, flexible, and multidimensional framework for capturing patient-reported health outcomes. Generic quality-of-life questionnaires, most commonly the Short Form-36 Health Survey (SF-36) and the EuroQol-5 Dimensions (EQ-5D/EQ-5D-3L), were frequently used to evaluate broader aspects of health status and well-being. While these measures enable comparison across different diseases, they are not specifically designed to detect psychiatric symptoms.

Finally, some studies used the Health Assessment Questionnaire (HAQ). This instrument primarily measures functional disability in daily living activities and therefore does not directly assess psychiatric outcomes, although impairment in function may indirectly reflect the psychosocial burden of RA.

### 3.2. TNF-α Inhibitors

A total of 10 studies evaluated TNF-α inhibitors, including 5 biologic agents: infliximab (IFX) [15,23,24], etanercept (ETN) [25,26,27,28,29], adalimumab (ADL) [16], golimumab (GLM) [15], and certolizumab pegol (CZP) [30]. Table 3 provides an overview of clinical studies assessing the neuropsychiatric effects of TNF-α in RA patients. It includes details on the agents used, study populations, and administration routes, offering context for the observed associations between immune modulation and improvements in depressive symptoms.

Eight of these studies reported clear improvements in depressive symptoms or quality of life as measured by validated scales (HADS, SDS, SF-36, EQ-5D-3L, PROMIS). The two studies [24,30] found no significant improvement, particularly in subgroups with comorbid somatization or insufficient inflammatory control.

Early RCTs demonstrated the positive impact of ETN on mental health outcomes. Significant improvement in depressive symptoms and quality of life (measured by MOS SF-36) in patients receiving ETN compared to placebo was reported [25]. Similar findings were observed in subsequent studies evaluating etanercept combined with methotrexate (MTX). Machado et al. [27] and Bae et al. [26] demonstrated that ETN + MTX significantly improved depressive symptoms, as assessed by HADS, outperforming conventional DMARD combinations.

Kekow et al. [28,29] showed in double-blind trials that ETN + MTX was superior to MTX alone in improving patient-reported outcomes (PROs), including reductions in depressive symptoms, with sustained effects observed over 104 weeks of follow-up.

Similarly, Miwa et al. [23] reported that IFX, a chimeric monoclonal antibody, significantly improved depressive symptoms (measured by the SDS scale) compared to MTX in a pilot study. However, a subsequent open-label study [24] found no significant differences between IFX and MTX in depression outcomes, as measured by the HAM-D scale.

The Phase 4 AWARE study [15], a large prospective observational study, further confirmed improvements in mood and PROMIS measures in patients treated with GLM or IFX over 46–52 weeks. Additionally, Hsieh et al. reported that ADL treatment improved depressive symptoms, assessed via EQ-5D-3L, in biologic-naïve RA patients over 24 weeks.

Furthermore, Curtis et al. [30] highlighted that CZP was less effective in RA patients presenting with the somatization comorbidity phenotype (SCP), who exhibited a lower likelihood of treatment response and a higher incidence of adverse events.

### 3.3. IL-6/IL-6R Inhibitors

Among the six studies evaluating IL-6 pathway inhibitors, five focused on agents targeting the IL-6R—tocilizumab (TCZ) [17,31,32,33] and sarilumab (SARI) [18]. In contrast, one study investigated sirukumab (SRK), a monoclonal antibody that directly binds to IL-6 itself [34].

These studies collectively support the potential psychiatric and immunological benefits of modulating the IL-6 signaling axis, with receptor blockade showing particular promise in patients with elevated IL-6 activity (see Table 4).

All 6 studies reported improvements in depressive symptoms, fatigue, and cognitive dysfunction. RA patients with elevated baseline IL-6 levels derived greater benefits in terms of mood and quality of life improvements when they were treated with SRK or SARI compared to those treated with TNF-α inhibitors [18,34]. These findings suggest that IL-6 blockade may more directly target neuroimmune pathways implicated in mood disturbances.

TCZ, a humanized IgG1 monoclonal antibody targeting the IL-6R [35], was shown to effectively reduce depressive and anxiety symptoms, with a sustained downward trend observed in more than 65% of RA patients [17]. These results were corroborated in a cohort of patients with active RA treated in routine clinical practice [31].

A pooled analysis of five RCTs comparing TCZ with conventional DMARDs reported that depression severity (based on medical records, stratified by timing and confirmed by clinical investigators) was associated with reduced rates of clinical remission (measured by CDAI and SDAI), although no such association was found with inflammatory markers such as CRP [32]. Moreover, the study highlighted that pharmacological treatment with SSRIs/SNRIs did not improve the subjective components of disease activity or pain perception among depressed individuals.

In the ARATA project, patients stratified by depressive status (BDI-II) showed a reduced treatment response. They also reported a higher prevalence of adverse symptoms compared to non-depressed individuals [33].

In a double-blind RCT evaluating SRK, a monoclonal antibody directly targeting IL-6, Sun et al. reported positive effects on depressive and anhedonic symptoms [34]. A post hoc analysis revealed that, compared to placebo, patients randomized to SRK showed significant improvements in depression and anhedonia, with outcomes positively correlated with baseline soluble IL-6 receptor levels [34].

A subsequent post hoc analysis from a RCT comparing SARI and ADL evaluated the predictive role of baseline IL-6 levels and showed that patients with high baseline IL-6 reported worse functional PROs than those with lower levels. Interestingly, SARI demonstrated greater efficacy than ADL among patients with high IL-6 levels and an inadequate response or intolerance to prior DMARD therapy [18].

## 4. Discussion

This review highlights the potential psychiatric benefits of biological therapies, particularly TNF-α and IL-6 inhibitors, in patients with RA. Across the 16 analyzed studies, improvements in depressive symptoms, fatigue, and cognitive dysfunction were consistently reported alongside enhanced somatic disease control and patient-reported outcomes (PROs). These findings align with the growing recognition that systemic inflammation contributes not only to joint pathology but also to psychiatric symptoms through complex neuroimmune mechanisms.

### 4.1. TNF-α Inhibitors and Psychiatric Outcomes

TNF-α inhibitors were among the first biologic therapies to demonstrate effects extending beyond joint inflammation. Improvements in depressive symptoms and quality of life have been consistently observed, particularly when TNF-α inhibition was combined with methotrexate [26,27]. Similar effects have been reported in other autoimmune diseases, such as psoriasis and inflammatory bowel disease, where TNF-α blockade was associated with reductions in somatic and psychiatric symptoms, including fatigue, depressive symptoms, and anxiety [36,37,38,39,40].

Further evidence comes from studies in ankylosing spondylitis (AS), which reported reductions in depressive symptoms with TNF-α inhibitors. Infliximab and etanercept, when used as part of standard treatment, were associated with reduced depression and anxiety severity and improved quality of life [41,42]. Preclinical studies provide further support: in animal models of chronic mild stress, infliximab reduced depressive-like and anxiety-like behaviors [43,44]. Moreover, TNF-α inhibitors have been explored as adjunctive treatments in bipolar disorder, where infliximab showed benefits on depressive symptoms, cognitive function, and neuroinflammatory markers [45,46,47].

These findings suggest that TNF-α plays a critical role in immune pathways that contribute not only to peripheral inflammation but also to central mechanisms implicated in mood regulation and cognition. The psychiatric benefits of TNF-α inhibition likely stem from its capacity to reduce systemic and CNS inflammation, restore blood–brain barrier (BBB) integrity, attenuate neuroinflammatory signaling, and normalize neurotransmitter systems. Preclinical models corroborate these mechanisms, showing reductions in anhedonia and anxiety-like behaviors after TNF-α blockade [11,14].

However, not all studies reported uniform benefits. Some indicated limited effects, particularly in patients with somatization comorbidity phenotypes, highlighting the complex interplay between biological and psychological factors in RA [30]. In these subgroups, inflammatory pathways may play a less dominant role compared to primary psychiatric mechanisms, potentially limiting the psychiatric impact of TNF-α inhibition.

### 4.2. IL-6/IL-6R Inhibitors and Psychiatric Outcomes

IL-6 inhibitors, such as tocilizumab and sarilumab, appear to offer more targeted psychiatric benefits, particularly among patients with elevated IL-6 levels. These observations support the hypothesis that IL-6 plays a direct role in neuroinflammatory processes relevant to mood disorders [18,25]. Unlike TNF-α inhibitors, IL-6 blockade demonstrated psychiatric benefits even without significant reductions in peripheral markers like CRP, suggesting that central neuroimmune effects may be more critical than systemic inflammation alone [32]. Post hoc analyses further indicated that higher baseline IL-6 or soluble IL-6 receptor levels predicted better psychiatric outcomes following IL-6 inhibition, aligning with precision medicine approaches [33].

Experimental data provide further support. IL-6 can cross the BBB, activate glial cells, and amplify neuroinflammatory responses, thereby influencing mood regulation. Moreover, IL-6 impacts the hypothalamic–pituitary–adrenal (HPA) axis and modulates neurotransmitter systems, contributing to fatigue, anhedonia, and cognitive dysfunction [12]. By mitigating these processes, IL-6 inhibitors may offer benefits for mood and cognition beyond their anti-inflammatory effects in peripheral tissues.

### 4.3. Proposed Mechanisms Linking Biological Therapies to Psychiatric Outcomes

**Figure 3:**
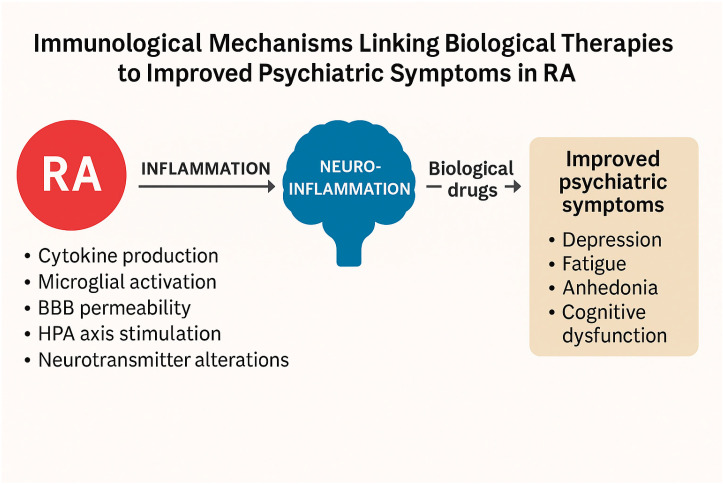
Immunological mechanisms linking inflammation in RA to neuropsychiatric symptoms and the effects of biologic therapies. Chronic systemic inflammation mediated by cytokines such as TNF-α and IL-6, contributes to blood–brain barrier dysfunction, neuroinflammation, HPA axis dysregulation, and altered neurotransmission, leading to psychiatric symptoms (depression, anxiety, fatigue, and cognitive impairment). Biological therapies targeting TNF-α and IL-6/Il-6R reduce systemic inflammation, which may alleviate neuroinflammatory processes and improve mental health outcomes.

The psychiatric improvements observed with TNF-α and IL-6 inhibitors are biologically plausible given the established roles of these cytokines in neuroimmune interactions (Figure 3). Chronic systemic inflammation affects the central nervous system (CNS) through multiple interconnected mechanisms:

#### 4.3.1. Blood–Brain Barrier (BBB) Dysfunction

Inflammation increases BBB permeability, allowing peripheral cytokines to influence the brain microenvironment [11,22]. Both TNF-α and IL-6 contribute to this process, enabling pro-inflammatory mediators to access the CNS.

#### 4.3.2. Microglial Activation and Neuroinflammation

Elevated TNF-α and IL-6 levels activate microglia, initiating sustained neuroinflammation and altering neurotransmission, particularly in circuits involved in mood regulation [21].

#### 4.3.3. Hypothalamic–Pituitary–Adrenal (HPA) Axis Dysregulation

Pro-inflammatory cytokines disrupt HPA axis function, leading to hypercortisolemia and its detrimental effects on mood, cognition, and energy balance [12,22].

#### 4.3.4. Kynurenine Pathway Activation

Inflammation increases the activity of indoleamine 2,3-dioxygenase (IDO), shifting tryptophan metabolism towards neurotoxic kynurenine metabolites linked to depressive symptoms and cognitive impairment [13,22].

#### 4.3.5. Direct Actions of IL-6

IL-6 can cross the BBB, activate glial cells, amplify neuroinflammatory cascades, stimulate the HPA axis, and alter neurotransmission [12].

## 5. Role of Biological Therapies

Biological therapies targeting TNF-α and IL-6 may mitigate these neuroimmune mechanisms through reductions in systemic and CNS inflammation, normalization of HPA axis activity, suppression of microglial overactivation, and restoration of neurotransmitter balance. These effects likely underpin the improvements in psychiatric symptoms observed with these treatments [21].

TNF-α inhibitors, such as infliximab, etanercept, and adalimumab, reduce systemic inflammation, restore BBB integrity, decrease neurotoxic metabolite production, and downregulate neuroinflammatory signaling. These mechanisms are thought to improve monoaminergic neurotransmission (serotonin, dopamine) and support neuroplasticity, alleviating depressive symptoms. Preclinical studies support these actions, showing reductions in anhedonia and anxiety-like behaviors after TNF-α blockade [11,14].

Similarly, IL-6 inhibitors such as tocilizumab or sarilumab reduce systemic cytokine levels, CNS inflammation, and HPA axis hyperactivity. Clinical studies indicate that patients with elevated IL-6 derive greater psychiatric benefit from IL-6 receptor inhibition [18,34]. Experimental models further confirm the antidepressant-like effects of IL-6 inhibition through normalization of neuroinflammatory markers and behavior [12]. Our observations are in line with previous reviews emphasizing the high prevalence and clinical importance of neuropsychiatric comorbidities in RA, and their potential link to inflammatory pathways [48].

### 5.1. Limitations

This review has several limitations that should be acknowledged. First, the included studies were heterogeneous with respect to design, sample size, duration of follow-up, and outcome measures, which limits comparability and precludes meta-analytic synthesis. The review was conducted as a narrative analysis, and although quantitative data are presented, the methodological variability across studies prevents systematic or pooled evaluation.

Second, psychiatric outcomes were typically secondary or exploratory endpoints rather than primary trial objectives, which reduces the robustness of the evidence. Mechanistic hypotheses linking cytokine modulation to neuropsychiatric outcomes remain biologically plausible but are largely based on indirect or preclinical data, warranting cautious interpretation.

Third, the heterogeneity of assessment tools further complicates interpretation. Validated psychiatric instruments such as HADS, BDI-II, HAM-D/HDRS, and SDS were used in some studies, whereas others employed PROMIS domains (e.g., depression, fatigue, pain interference, physical function) or generic quality-of-life measures (SF-36, EQ-5D). In addition, the HAQ, which primarily measures functional disability rather than psychiatric symptoms, was used in several trials. This variability across tools limits the comparability of outcomes and highlights the need for standardized psychiatric measures in future research.

Finally, publication bias cannot be excluded, as studies reporting null findings in psychiatric outcomes may be underrepresented in the available literature.

### 5.2. Conclusions

Biologic therapies targeting TNF-α and IL-6 in rheumatoid arthritis may provide dual benefits, improving both joint-related disease activity and neuropsychiatric symptoms such as depression, fatigue, and cognitive dysfunction. These effects are likely mediated through modulation of systemic and central inflammatory pathways, including blood–brain barrier integrity, microglial activation, and HPA axis regulation.

While the current evidence supports these benefits, most studies assessed psychiatric outcomes as secondary endpoints. Future research should focus on well-designed prospective trials with standardized neuropsychiatric assessments and biomarker-based stratification to confirm and optimize these findings.

Recognizing psychiatric symptoms as part of the disease burden in RA opens the door to more holistic treatment approaches, where targeting immune pathways can contribute to improved mental and physical health outcomes.

### 5.3. Clinical Implications

Incorporating psychiatric symptom screening (Validated psychiatric tools—PHQ-9 or HADS) and considering neuroinflammatory profiles may help guide the selection of biologic therapy in RA, particularly in patients with comorbid depression or fatigue.

## Figures and Tables

**Table 1:** Summary of biologic agents targeting TNF-α and IL-6 pathways in rheumatoid arthritis, including origin, class, molecular structure, and mechanism of action *.

	Generic Name	Trade Name Original, Biosimilars	Group	Molecule Type	Mechanism of Action
ADL	Adalimumab	Humira, Amgevita, Hyrimoz, Idacio, Hulio, Amsparity, Imraldi	anti-TNF	Fully human monoclonal IgG1 antibody	Neutralizes TNF-α (soluble and membrane-bound forms); blocks p55/p75 receptors
CZP	Certolizumab Pegol	Cimzia	anti-TNF	Humanized Fab’ fragment conjugated with PEG	Neutralizes TNF-α; lacks Fc-mediated effects
IFX	Infliximab	Remicade, Inflectra, Remsima, Flixabi, Zessly	anti-TNF	Chimeric IgG1 monoclonal antibody (human-mouse)	Blocks TNF-α; inhibits inflammatory pathways
ETN	Etanercept	Enbrel, Benepali, Erelzi	anti-TNF	Fusion protein: p75 TNF receptor + Fc IgG1	Binds TNF-α and TNF-β; acts as a decoy receptor
GLM	Golimumab	Simponi (s.c.), Simponi Aria (i.v.)	anti-TNF	Fully human monoclonal IgG1 antibody	Blocks TNF-α; inhibits inflammatory pathways
SRK	Sirukumab	None (discontinued, never approved)	anti-IL-6	Fully human monoclonal IgG1 antibody	Neutralizes IL-6 (the cytokine, not the receptor)
SARI	Sarilumab	Kevzara	anti-IL-6R	Fully human monoclonal IgG1 antibody	Blocks IL-6 receptor (soluble and membrane-bound)
TCZ	Tocilizumab	RoActemra (EU), Actemra (USA)	anti-IL-6R	Humanized monoclonal IgG1 antibody	Blocks IL-6 receptor (soluble and membrane-bound)

* Only biologic drugs used in RA with documented effects on TNF-α or IL-6 pathways are included. Other biologics, such as rituximab (anti-CD20) or abatacept (T-cell costimulation inhibitor), were excluded as they are beyond the scope of this analysis focusing on neuropsychiatric outcomes. Abbreviations: ADL, adalimumab; CZP, certolizumab pegol; IFX, infliximab; ETN, etanercept; GLM, golimumab; SRK, sirukumab; SARI, sarilumab; TCZ, tocilizumab; TNF, tumor necrosis factor; IL-6, interleukin-6; IL-6R, interleukin-6 receptor; IgG1, immunoglobulin G1; Fab’, antigen-binding fragment; PEG, polyethylene glycol; Fc, crystallizable fragment of antibody; s.c., subcutaneous; i.v., intravenous.

**Table 2:** Psychiatric assessment tools were used across the included studies.

Study	Validated Psychiatric Tools	PROMIS Domains	Generic QoL Measures	Notes
Miwa et al., 2014 [23]	SDS	-	-	
Miwa et al., 2016 [24]	HAM-D	-	-	
Mathias et al., 2000 [25]	-	-	SF-36	
Bae et al., 2013 [26]; Machado et al., 2014 [27]; Kekow et al., 2010/2011 [28,29]	HADS	-	-	
Bingham et al., 2023 [15]	-	Depression; Fatigue; Pain Interference; Physical Function	-	
Hsieh et al., 2023 [16]	-	-	EQ-5D-3L	
Curtis et al., 2017 [30]	-	-	-	Clinical analysis: SCP affected response
Tiosano et al., 2020 [17]	HDRS	-	-	
Harrold et al., 2017 [31]	-	-	EQ-5D	
Manning-Bennett et al., 2022 [32]	-	-	HAQ (functional measure)	Diagnosis from medical history. Pain VAS; remission via CDAI/SDAI; antidepressant use as covariate
Behrens et al., 2023 [33]	BDI-II	-	-	
Sun et al., 2017 [34]	PDMA	-	SF-36	
Strand et al., 2020 [18]	-	-	SF-36	

SDS—Self-Rating Depression Scale; HAM-D/HDRS—Hamilton Depression Rating Scale; HADS—Hospital Anxiety and Depression Scale; BDI-II—Beck Depression Inventory-II; PROMIS—Patient-Reported Outcomes Measurement Information System; PDMA—Prevalent Depressed Mood and Anhedonia; SF-36—Short Form Health Survey 36; EQ-5D/EQ-5D-3L—EuroQol 5 Dimensions questionnaire; CDAI—Clinical Disease Activity Index; SDAI—Simple Disease Activity Index; SCP—Somatization Comorbidity Phenotype; HAQ—Health Assessment Questionnaire (Disability Index, functional status).

**Table 3:** Studies assessing the impact of TNF-α inhibitors on depression and quality of life in RA patients.

Study	Study Design (Treatment Length)	Biologic Agent	Measure	Outcome
Miwa et al., 2014 [23]	Pilot study (30 weeks)	IFX (n = 34) vs. MTX (n = 42)	SDS	IFX significantly improved depression vs. MTX
Miwa et al., 2016 [24]	Open-label cohort (6 months)	IFX (n = 60) vs. MTX (n = 53)	HAM-D	No significant difference between IFX and MTX
Bringham et al., 2023 [15]	Observational Phase 4 AWARE (52 weeks)	GAL (n = 685) vs. IFX (n= 585)	PROMIS	Improvement in all PROMIS domains incl. depression
Curtis et al., 2017 [30]	RCT Phase 4 PREDICT (52 weeks)	CZP (n = 733)	Clinical data	SCP: lower treatment response, more AEs
Mathias et al., 2000 [25]	RCT Phase 3, double-blind (6 months)	ETN (n= 76) vs. placebo (n = 80)	SF-36 MOS	ETN > placebo in improving depressive symptoms
Bae et al., 2013 [26]	Open-label, multicentre (16 weeks)	ETN + MTX (n = 197) vs. DMARDs +MTX (n = 103)	HADS	Greater improvements in ETN + MTX group (HADS)
Machado et al., 2014 [27]	Open-label, randomized (24 weeks)	ETN + MTX (n = 281) vs. DMARDs + MTX (n = 142)	HADS	Improvements observed in ETN + MTX depressive domains
Kekow et al., 2010 [28]	RCT double-blind, COMET 104 weeks	ETN + MTX (n= 274) vs. MTX (n= 268)	HADS	ETN + MTX better than MTX alone in PROs
Kekow et al., 2011 [29]	RCT double-blind, COMET * 104 weeks	ETN + MTX vs. MTX	HADS	Clinical remission reduced depressive symptoms
Hsieh et al., 2023 [16]	Observational 24 weeks	ADL (n = 100)	EQ-5D-3L	Improvements from baseline to weeks 12, 24

Abbreviations: IFX, infliximab; GAL, golimumab; CZP, certolizumab pegol; ETN, etanercept; ADL, adalimumab; MTX, methotrexate; DMARDs, disease-modifying anti-rheumatic drugs; SDS, Self-Rating Depression Scale; HAM-D, Hamilton Depression Rating Scale; PROMIS, Patient-Reported Outcomes Measurement Information System; SF-36 MOS, Medical Outcomes Study 36-Item Short Form Survey; HADS, Hospital Anxiety and Depression Scale; EQ-5D-3L, EuroQol 5 Dimensions 3 Level questionnaire; RCT, randomized controlled trial; AEs, adverse events; PROs, patient-reported outcomes. * Note1: The studies by Kekow et al. (2010 and 2011) [28,29] are based on the same COMET trial population. Note2: The total sample includes 3027 participants across all TNF-α inhibitor studies. Among them, approximately 2211 patients received active treatment with a TNF-α inhibitor, either as monotherapy or in combination with methotrexate (MTX), compared to placebo, MTX alone, or standard care.

**Table 4:** Studies assessing the impact of IL-6 inhibitors on depression and quality of life in RA patients.

Study	Study Design/Duration	Biologic Agent (n)	Measures	Outcome
Tiosano et al., 2020 [17]	Observational 24 weeks	TCZ (n = 91)	HDRS	66% of patients achieved improvements in depressive domains.
Harrold et al., 2017 [31]	Observational cohort study 1 year	TCZ (n = 255)	EQ-5D	20% to 36% of patients achieved improvements in depressive state.
Manning-Bennett et al., 2022 [32]	5 RCT	TCZ vs. DMARDs (n = 5502)	Clinical	Comorbid depression was associated with less frequent remission (CDAI and SDAI)
Behrens et al., 2021 [33]	Observational ARATA 52 weeks	TCZ (n = 1300)	BDI-II	Patients achieved improvements in DAS-28 and PROs; however, patients with depression presented lower response and higher adverse event rates.
Sun et al., 2017 [34]	Post hoc analysis RCT 24 weeks	sirukumab vs. siltuximab (n = 176)	PDMA includinngSF-36	Baseline solute IL-6 receptor levels predicted mental health benefit. The improvement in depressive state by sirukumab correlated positively with the baseline solute IL-6R levels.
Strand et al., 2020 [18]	Post hoc analysis RCT MONARCH phase 3, and they were treated for 24 weeks	SARI or ADL (n = 148)	SF-36	IL-6 blockade > TNF-α in QoL gains no difference in the mental state. High baseline IL-6 levels better improvements in physical domains with SARI compared to ADL.

Abbreviations: HDRS (Hamilton Depression Rating Scale); EQ-5D (EuroQol-5 dimensions-5); DMARDs (Disease-Modifying Antirheumatic Drugs); CDAI (Clinical Disease Activity Index); SDAI (Simple Disease Activity Index); PROs (Patient-Reported Outcomes); BDI (Beck Depression Inventory), PDMA (Prevalent Depressed Mood and Anhedonia); SF-36 (Short Form Survey); n-number of participants. Note: The total sample includes 7472 participants across all IL-6 or IL-6R inhibitor studies. Approximately 4609 patients received active treatment with an IL-6 pathway inhibitor, either alone or in combination with MTX, and were compared to MTX, placebo, or conventional DMARDs.

## Data Availability

No new data were created or analyzed in this study. Data sharing is not applicable to this article.

## Abbreviations

AA	Alopecia Areata
ADA/ADL	Adalimumab
AE	Adverse Events
AM	Amplitude Modulation
AS	Ankylosing Spondylitis
BDI	Beck Depression Inventory
BBB	Blood–Brain Barrier
CD	Crohn’s Disease
CNS	Central Nervous System
CRP	C-Reactive Protein
CT	Computed Tomography
CZP	Certolizumab Pegol
DAS28	Disease Activity Score 28
ETA	Etanercept
EQ-5D	EuroQol 5 Dimensions Questionnaire
FACIT-F	Functional Assessment of Chronic Illness Therapy-Fatigue
FDA	Food and Drug Administration
GLM	Golimumab
HAQ	Health Assessment Questionnaire
HADS	Hospital Anxiety and Depression Scale
HPA	Hypothalamic–Pituitary–Adrenal
IBD	Inflammatory Bowel Disease
IDO	Indoleamine 2,3-Dioxygenase
IgG	Immunoglobulin G
IL	Interleukin
IL-6	Interleukin-6
IL-6R	Interleukin-6 Receptor
mAb	Monoclonal Antibody
MRI	Magnetic Resonance Imaging
MTX	Methotrexate
PCS	Physical Component Summary
PHQ-9	Patient Health Questionnaire-9
PRISMA	Preferred Reporting Items for Systematic Reviews and Meta-Analyses
PROs	Patient-Reported Outcomes
QoL	Quality of Life
RA	Rheumatoid Arthritis
SARI	Sarilumab
SF-36	Short Form Health Survey 36
SRK	Sirukumab
TCZ	Tocilizumab
TNF	Tumor Necrosis Factor
TNF-α	Tumor Necrosis Factor-alpha
UC	Ulcerative Colitis
VAS	Visual Analog Scale
WOMAC	Western Ontario and McMaster Universities Osteoarthritis Index
